# New Directions in Migraine Pathophysiology: The Glymphatic System and PACAP

**DOI:** 10.3390/life16050767

**Published:** 2026-05-03

**Authors:** Dan Iulian Cuciureanu, Cătălina Elena Bistriceanu, Georgiana-Anca Vulpoi, Victor Constantinescu, Diana Laura Blajuta, Ana-Maria Nădejde, Florina Antochi, Adina-Maria Roceanu

**Affiliations:** 1 Neurology Department, Faculty of Medicine, University of Medicine and Pharmacy “Grigore T. Popa”, 16 Universitatii Street, 700115 Iasi, Romania; 2 Neurology Department I, “Prof. Dr. N. Oblu” Emergency Clinical Hospital, 2 Ateneului Street, 700309 Iasi, Romania; aursulesei_ana_maria@yahoo.com; 3 Elytis Hospital Hope, 43A Gheorghe Saulescu Street, 700010 Iasi, Romania; 4 Arcadia Hospitals and Medical Centers, 38 Sarariei Street, 700116 Iasi, Romania; 5 Department of Neurology, Rehabilitation Hospital, 700661 Iasi, Romania; 6 Faculty of Medicine, University of Medicine and Pharmacy “Grigore T. Popa”, 16 Universitatii Street, 700115 Iasi, Romania; 7 Doctoral Study University of Medicine and Pharmacy, Grigore T. Popa Iași, Romania, 16 Universitatii Street, 700115 Iasi, Romania; 8 Neurology Department, University Emergency Hospital, 050098 Bucharest, Romaniaamr2012mar@gmail.com (A.-M.R.)

**Keywords:** migraine, neuropeptides, PACAP, glymphatic system

## Abstract

Migraine severely impacts the quality of life of young adults. During the past few years, many studies have been done regarding the pathophysiology of this condition. There has been intense debate regarding CGRP, but research is still underway about the glymphatic system and PACAP. This review provides an overview of the current literature in this area of migraine pathophysiology. The inflammatory mediators and neuropeptides that activate trigeminovascular pathways can be accumulated during migraine attacks as a result of a failure of glymphatic clearance. Neuroinflammation, CGRP, CSD, and sleep have all been linked to the glymphatic system and migraine. In this article, we also discuss the latest hypotheses regarding the PACAP pathway in the neurophysiology of migraine. Additionally, recent research suggests that glymphatic dysfunction could enhance PACAP-mediated signaling. This article will explore possible correlations between these mechanisms and migraine pathophysiology.

## 1. Introduction

Globally, migraine is the sixth most disabling disease, with complex mechanisms that are still not fully understood [1]. A study that analyzes the data related to migraine (prevalence, incidence, and DALYs (disability-adjusted life-years) from 204 countries and territories over a period of more than 32 years) from the GBD (Global Burden of Disease) 2021 report concludes a substantial increase in migraine prevalence in the interval 15–39 years [2]. According to some authors, there are several comorbidities of migraine, such as psychiatric, epilepsy or microbiota impairment, which are explained by a variety of pathophysiologic mechanisms. By focusing on these pathologies, headache recurrence may be prevented, and quality of life may increase [3]. Because migraines cause substantial disability in young adults, the identification of new etiopathogenic links opens new approaches to the treatment and understanding of this condition.

During the past few years, research on the role of the glymphatic system in migraine pathology has been steadily increasing. There has been considerable research on the role of the lymphatic system in many neurological diseases, but its role in migraine is still being studied. Preliminary studies have been completed on mice injected with nitroglycerine (C57/BL6). The suppression of glymphatic function by AQP4 blockers (TGN-020) has been observed to exacerbate physical and psychological manifestations of migraine in these mice [4].

Despite this fact, the nitroglycerin mouse model does not exactly mimic human migraine pathophysiology, since it represents a provoked state, compared to humans with multifactorial migraine attacks (hormones, sleep patterns, genetics, environmental factors, etc.).

Since AQP4 does not play a major role in migraine pathology in humans, results from animals must be extrapolated cautiously due to species differences and the more complex astrocyte signaling in humans.

For a long time, it was thought that there was no lymphatic system in the brain. In an attempt to answer the question of how the brain removes soluble interstitial wastes, the mechanism of clearance via the glymphatic system was proposed in 2012 [5].

A study has demonstrated that CSD (cortical spreading depression) causes a major impact on paravascular space and that the glymphatic flux is aligned with and influenced by CSD. As a result of this phenomenon, the paravascular space and penetrating cortical vessels rapidly closed in the animal model for a few minutes and then gradually recovered for 30 min [6]. The aim of this review is to present the latest literature regarding mechanisms related to the role of the glymphatic system in migraine pathophysiology.

The endogenous neuropeptides, such as calcitonin-gene related peptide (CGRP), pituitary adenylate cyclase-activating peptide (PACAP), have become increasingly studied in recent decades. In particular, PACAP is being studied in patients who do not respond to monoclonal antibodies against anti-CGRP [7]. Our goal is to provide an overview of the data related to this neuropeptide as well as the latest studies linking it to migraine mechanisms.

We searched in PubMed/MEDLINE and ScienceDirect English-language human and animal studies using MeSH (Medical Subject Headings) terms like “glymphatic” AND “migraine” AND/OR “physiopathology”; “PACAP” AND “migraine” AND/OR “physiopathology; and “glymphatic” AND “PACAP” AND “migraine”. Among the studies we reviewed, there were randomized controlled trials, observational studies, and systematic reviews published between 1991 and 2026.

## 2. The Anatomy of the Glymphatic System

The glymphatic system is described as a network of perivascular spaces filled with cerebrospinal and interstitial fluid. This system consists of penetrating and pial vessels that function to eliminate metabolic waste products and fluids from the interstitial space [8]. The glymphatic system, along with cerebrospinal fluid and interstitial fluid, is considered part of the brain network responsible for clearing metabolic wastes and transporting water [9]. As first described in 2012, the glymphatic system is composed of glial cells and barrier systems that modulate circulation and production of neurofluid [10]. There are several anatomical structures comprising this system, including the perivascular space and the aquaporin-4 (AQP4) channel [11].

The perivascular space (PVS) surrounds arterioles, capillaries, and venules, and appears on MRI as parallel to and spatially correlated with perforating vessels and perpendicular to the brain’s surface [12]. An outer limit of the perivascular space is the glia limitans, whereas an inner limit is the endothelial basement membrane. The basal perforating arterioles are surrounded by two layers of leptomeninges, the inner layer adherent to the arteriolar wall, and the outer layer extending from the pia mater [13]. As well as being connected to the blood–brain barrier (BBB), this space is also connected to the cerebrospinal fluid (CSF) circulation and the perineural lymphatic drainage [14]. PVSs contain CSF between vessels and pia mater; arterial pulses and CSF pressure gradients determine the flow of CSF into arterial PVSs [15]. The subarachnoid CSF blends with the interstitial fluid and is cleared along specific paravenous pathways, including those of the internal cerebral and the caudal vein [16]. In the perivascular space, astrocyte foot processes surround blood vessels and form low-resistance tubes. The PVS consists of an inner cylinder, which represents the cerebral vascular wall, and an outer cylinder, which represents the glial boundary that surrounds blood vessels or ends of astrocytes that penetrate arterioles [17].

An important role of AQP4 is to facilitate CSF influx across the vascular endpoint of astrocytes, for waste clearance. Astrocytic endfeet are highly enriched in aquaporin (AQP) channels, which facilitate passive water transport across cell membranes [18].

AQP4 function was manipulated by administering AER-271 by inhibiting glymphatic influx as measured by CSF tracers infused into the cisterna magna, as well as interstitial fluid volume increases as measured by diffusion-weighted MRI in animal studies. In agreement with the authors’ conclusion, the aquaporin-4 inhibitor AER-271 acutely reduces glymphatic flow similarly to the glymphatic decrease caused by genetic deletion of aquaporin-4 [19].

The meningeal lymphatics are thought to be the source of the negative pressure that allows glymphatic flow. They absorb the subarachnoid CSF, which drives soluble brain waste to the extracranial lymph nodes. Perivascular lymphatic drainage represents the primary route for antigens from the brain to cervical lymph nodes [20].

**Figure 1:**
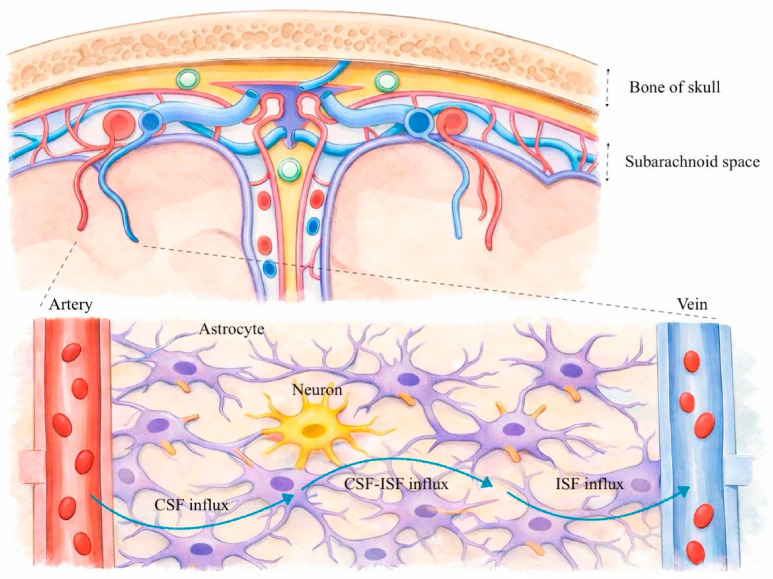
The anatomy of the glymphatic system: cerebral spinal fluid (CSF), interstitial fluid (ISF), perivascular spaces, vessels, glial cells (drawn by Diana Laura Blăjuță).

As a conclusion from the data presented above, CSF from the subarachnoid space enters the brain parenchyma through the perivascular spaces surrounding the penetrating arteries; aquaporin water channel-4 (AQP4) located in astrocyte terminal legs assists it in entering the interstitial spaces. CSF mixed with interstitial fluid and residual interstitial substances is drained through the perivenous spaces along the veins to enter the CSF circulation (either in the ventricles or subarachnoid space, or it may pass through the bloodstream by specific transport mechanisms at the blood–brain barrier). Through arachnoid granulations, cranial nerves, and nasal lymphatics, debris from the CSF compartments escapes further from the skull [5]. Figure 1 shows a schematic representation of some of the steps involved in this process.

In several neurological disorders, including neurodegenerative diseases, the glymphatic system has been studied. Following this, we will present recent data from the literature regarding migraine and the glymphatic system.

## 3. Glymphatic System in Migraine

The glymphatic system plays a crucial role in removing waste products from the brain, like the lymphatic system at the periphery of the body. There is a well-known association between impaired glymphatic function and neurodegenerative diseases through abnormal protein accumulation, but migraine has also been linked to impaired glymphatic function. At least four perspectives can be used to study the connection between the glymphatic system and migraine: neuroinflammation, CGRP, CSD, and sleep [21].

### 3.1. Findings Related to Cortical Spreading Depression

Mice injected with nitroglycerin (C57/BL6) were used for preliminary studies. An AQP4 blocker (TGN-020) aggravated migraine pathophysiological changes in mice by suppressing glymphatic function [4].

Also, animal models have been used to study the connection between the glymphatic system and the cortical spreading depression (CSD) phenomenon. In the animal model, CSD led to a rapid and almost complete obstruction of the paravascular space and of penetrating cortical arteries and veins, which gradually recovered after 30 min. A new mechanism for regulating glymphatic flow could be defined by this closure. The CSD has been shown to have a dramatic effect on the size of the paravascular space, and glymphatic flow is delayed and slowed by the CSD [6]. It should be noted, however, that many mechanisms related to animal models of CSD do not completely overlap with human migraine cascades. The dural puncture in mice could impair the glymphatic system in this study, and the measurement in anesthetized mice could also be biased since animals asleep have a different glymphatic flux than awake animals. Other approaches to this subject could include neuroimaging biomarkers for perivascular spaces (e.g., DTI-APLS = diffusion tensor imaging along the perivascular space). As a result, glymphatic dysfunction can be defined based on imaging-based markers. However, the differences between the in vivo and in vitro glymphatic system results could be improved.

As a result of migraine aura, there is an impairment of ion homeostasis, cellular edema, and multiphasic changes to blood flow, which are linked to paravascular closure. As previously mentioned, PVS serves as an outflow route for interstitial fluid and soluble substances, including inflammatory and excitatory substances (which are more prevalent following CSD). The closure of PVS may be mediated by astrocytic astrocyte head swelling caused by increased expression of aquaporin water channel protein aquaporin-4 (AQP4) [22].

In another animal model study, glutamate overflow was determined by the duration of Ca2+ signals in astrocytic cells in the advanced phase of CSD. AQP4-dependent swelling evoked by an increase in extracellular K+ seemed to be responsible for the rise in extracellular glutamate. It can be concluded, therefore, that glial cells contribute significantly to CSD’s extracellular glutamate [23]. A significant aspect of this study was that it was conducted on awake mice, which avoided the effects of anesthetics on fluid dynamics. This study suggests AQP4 plays an important role in CSD through ion shifts, glutamate imbalances, and astrocyte swelling. The extended role of AQP4 in the brain and glymphatic system poses difficulties in putting it into practice in humans, and further research is required (on AQP4 and astrocyte swelling as potential therapeutic targets in migraine).

In the early stages of CSD, neurons receive energy support from astrocytes so that they can remove K+ and glutamate from synaptic gaps. As the energy demand exceeds the astrocyte’s capacity to compensate, they release large quantities of lactic acid and exacerbate hypoxia. CSD persists as a result of astrocytes’ endfoot swelling, causing perivascular space closure, slowing the glymphatic system, and exacerbating neuroinflammatory processes [24].

During CSD, potassium, glutamate, and ATP levels increase, and there is a cascade of inflammation that results in the activation of microglia and astrocytic cells, as well as induced nitric oxide synthase and COX-2 production in the brain parenchyma. As a result of the transient blockade of outflow of excitatory or inflammatory chemicals from the interstitial space following CSD episodes, clearance of these macromolecules may be temporarily suppressed, explaining the development of hyperexcitability and structural changes in the cortical region [25].

### 3.2. Neuroinflammation, CGRP and Glymphatic System in Migraine

Studies have tried to clarify whether there are immune cells in meninge, including lymphocytes and mast cells, which can be transported to the glymphatic system. The release and detection of proinflammatory cytokines (i.e., IL-6, IL-10, MCP-1, TNFα, IL-12p70, and IFNγ) under basal conditions has been reported by some authors, which confirms the local source of inflammatory stimulus causing migraines [26].

A murine model of chronic migraine and two genetic models of CGRP signaling insufficiencies, Ramp1 systemic deficiency and lymphatic-specific CalCRL insufficiency, have been used to clarify the role of meningeal lymphatics in chronic migraine pain and pathophysiology. The injection of CGRP directly into the cisterna magna inhibited CSF drainage and reduced the permeability of meningeal lymphatic vessels. Based on the results of this study, lymphatics play a previously unrecognized role in chronic migraine, whereby CGRP signaling primes lymphatic vessel–immune interactions and reduces CSF efflux [27].

In the context of neuroinflammation, the glymphatic system is involved in the removal of reactive oxygen species (ROS) and inflammation caused by ROS. A dysfunctional glymphatic system may lead to increased releases of proinflammatory cytokines, such as tumor necrosis factor -α, interleukin-1β, and hypoxia-induced factor-1α, released by microglia. The accumulation of ROS and pro-inflammatory cytokines exacerbates neuronal degeneration and astrocyte damage, worsening glial dysfunction. An increase in pain sensitivity can be caused by proinflammatory cytokines activating nociceptors [28].

As a result of damage to the glymphatic system, proinflammatory cytokines and ROS may accumulate, resulting in damage to the astrocytes. Proinflammatory cytokines can further aggravate nociceptive stimuli by overactivating neurons and nociceptors. The possibility of neuroinflammation underlying glymphatic system dysfunction is discussed in migraine pathophysiology [8].

It has been shown that CGRP plays a central role in migraine. By releasing neuropeptides (including CGRP) from the trigeminal nerve fibers, the meninges initiate and maintain ‘neurogenic inflammation.’ According to animal studies, MCP-1, CGRP, and IL12-p70, which are implicated in migraine pathology, are shifted locally when the meningeal lymphatics develop defectively [26].

The activation of dural afferent neurons releases several neuropeptides, including calcitonin gene-related peptide (CGRP) and pituitary adenylate cyclase-activating polypeptide (PACAP). Multiple immune cells can be activated by these neuropeptides, and more mediators are released, further modulating the activity of dural afferent neurons [29]. Neuroinflammation can thus be conceptualized as an increase in serum levels of CGRP and certain proinflammatory cytokines.

By activating the trigeminal afferents innervating the penetrating cerebral arteries, CGRP may be released into the perivascular space and transported via the glymphatic system. There is a possibility that CGRP may collect in lymphatic vessels within the superior sagittal sinus. According to recent evidence, perivascular spaces collapse during experimental cortical spreading depression, which results in migraine auras. It may be argued that the glymphatic system serves as an additional sink for CGRP from this perspective [30].

Trigeminal C-fibers release CGRP during a migraine attack, causing the vasodilation of dural venous sinuses that are in contact with dural meningeal lymphatic vessels (MLVs). Providing the base for the efflux of CSF and waste produced by neural cells, these structures bind the central and peripheral immune systems together. Thus, it is hypothesized that CGRP/CGRP receptor signaling in MLVs regulates brain fluid drainage and neuroimmune inflammation during migraines [31].

While the mechanism of CGRP peripheral action is unclear, it is supported by the fact that CGRP administration triggers attacks without crossing the blood–brain barrier. It is believed that the nociceptive signaling from the dura mater plays an important role in headache attacks [32].

### 3.3. Glymphatic System and Sleep in Migraine

According to one hypothesis, sleep deprivation reduces glymphatic system clearance, resulting in an accumulation of headache-related substances in the interstitial fluid. It is possible that these conditions may lower the threshold for headaches, contributing to the possibility that migraines may become chronic [33].

Glymphatic system activity is particularly active during sleep in order to eliminate potentially toxic neuronal waste products that accumulate during wakefulness. It has been shown that sleep deprivation reduces glycogen breakdown, which contributes to CSD, raises extracellular K+ levels, activates inflammatory pathways, and impairs glymphatic transport, leading to a lack of glucose or lactate transporters in the cortex, resulting in migraine pathophysiology [34].

Furthermore, norepinephrine (NE), which regulates glymphatic function, also plays an important role in migraines and sleep. In addition to regulating sleep–wake cycles and extracellular perivascular space volume, NE regulates glymphatic exchange. It is believed that waking causes NE release, which stops glymphatic function and increases fluid transport resistance. Researchers have found that using NE on the dura mater causes rats to exhibit headache-like behaviors [35].

Poor sleep quality or sleep deprivation in chronic migraine may result in hypoactivation of the glymphatic system, which may lead to headache maintenance. Glymphatic dysfunction has been proposed to contribute to several types of headaches, and these ideas have been explored in recent years [25]. Also, AQP4 contributes to the function of the glymphatic system and regulates sleep–wake cycles in humans. In sleep-deprived people experiencing migraine attacks, AQP4 depolarization remains still unknown as a potential trigger [33].

Our next topic will focus on PACAP’s role in migraine pathophysiology, along with the latest research on this topic.

## 4. The Role of Vasoactive Peptides in Migraine’s Pathophysiology

Neuropeptides may contribute to migraine pathophysiology by modulating synaptic transmission in the central nervous system (CNS). They may also act in peripheral tissues, influencing cellular activities. In the brain, they act as neuromodulators, and in the peripheral circulation, they act as signaling molecules. As a result of these characteristics, neuropeptides can modify migraine sensory perception. Among the neuropeptides involved in migraine pathophysiology, CGRP and PACAP share many characteristics, which may underlie their mechanisms in migraine [36]. PACAP belongs to the glucagon/secretin superfamily. In addition to PACAP, other peptides like it include vasoactive intestinal peptide (VIP), which shares 68% homology [7].

PACAP is present in peripheral structures relevant to the pathogenesis of primary headaches, including migraine, such as the trigeminovascular system, dural nerve fibers, cerebral vessels, the parasympathetic sphenopalatine ganglion, and the otic ganglia [37]. PACAP receptors, particularly PAC1, as well as VPAC1 and VPAC2, have been identified in several peripheral structures, including blood vessels, autonomic ganglia, sensory nerve endings, and immune cells such as mast cells [38]. Through this localization, the PACAP system may contribute to vasodilation and neurogenic inflammation, promoting mast cell degranulation and the release of inflammatory mediators [39]. At the peripheral level, PACAP has been reported to exert antinociceptive effects [40]. In contrast, PACAP in the CNS appears to have a pronociceptive role, as suggested by studies in PACAP knockout mice, which indicate a possible involvement in central sensitization [41].

At the level of the CNS, PACAP fibers and PAC1 receptors are widely distributed, with particularly high expression in the hypothalamus and supraoptic nucleus. Other sites include the dorsal root ganglia, where PACAP may modulate nociception, and the pontine superior salivatory nucleus, which regulates preganglionic parasympathetic neurons and may contribute to somatosensory and autonomic manifestations [42]. PACAP-related activation has also been described in brainstem nuclei, including the periaqueductal gray, locus coeruleus, and raphe nuclei, which modulate central trigeminovascular neurons and descending pain-control circuits [43]. PACAP signaling may be relevant within the trigeminocervical complex, particularly in the trigeminal nucleus caudalis, as well as in brain regions involved in nociception and autonomic regulation, as discussed above, but also in circadian control and stress responses [42]. PACAP may act as an important regulator of the circadian system through its role in retinohypothalamic tract signaling. It modulates the phase of the suprachiasmatic nucleus (SCN) via PACAP-R1 and cAMP-dependent pathways, suggesting that PACAP may stimulate cAMP production in SCN neurons after appropriate retinal input [44]. Moreover, PACAP/PAC1 signaling is essential for activation of the hypothalamic–pituitary–adrenal axis and may contribute to stress-related responses in regions such as the amygdala, raphe nuclei, and locus coeruleus [45].

Table 1 briefly summarizes the main differences between the central and peripheral effects of PACAP.

While migraine mechanisms are still poorly understood, clinical and preclinical studies over the past three decades have focused attention on CGRP neuropeptides. However, a significant proportion of migraine sufferers do not benefit sufficiently from preventive treatment with anti-CGRP antibodies, highlighting the need for novel therapeutic agents based on other pathophysiological mechanisms [39]. The evidence suggests that CGRP is not the only neuropeptide involved in migraine, and that other neuropeptides may also be involved in the pathophysiology of migraine, such as PACAP [25,39].

CGRP plays a central role in migraine pathophysiology, but it does not fully account for all migraine mechanisms. In this context, PACAP appears to exert a related yet distinct role, making PACAP and its receptors promising therapeutic targets that may complement current CGRP-based migraine treatments [39].

### 4.1. PACAP and Migraine

PACAP and its receptors are widely expressed throughout the body across various species, with particularly high concentrations in the CNS. They are notably present in key migraine-related structures, such as the hypothalamus and trigeminal ganglia, highlighting their potential role in migraine mechanisms [39]. The trigeminovascular system, comprising the trigeminal nerve, cerebral vasculature, and central structures within the brainstem and spinal cord, plays a pivotal role in migraine pathophysiology. Afferents of the trigeminal nerve innervate the cerebral vascular system peripherally and project to the trigeminal ganglia (TG). PACAP is expressed throughout the trigeminovascular system and in higher brain regions involved in migraine pain processing [46]. Three G protein-coupled receptors (PAC1, VPAC1, VPAC2) mediate the effects of PACAP38 [47]. The PAC1 receptor is selectively activated by PACAP, whereas the VPAC1 and VPAC2 receptors respond to both PACAP and VIP [39]. In addition to the complexity of the PACAP receptor family, a lack of well-characterized molecular tools and the absence of animal models of migraine have made it difficult to determine which PACAP receptor(s) are involved in migraine pathophysiology [46]. Among the three receptors, PAC1 is considered the most likely pathophysiological target of PACAP in migraine. In this context, antibodies specifically targeting the PAC1 receptor may be essential both for clarifying the role of PACAP in migraine pathophysiology and for determining whether the PACAP receptor system could represent a therapeutic target in other forms of craniofacial pain [46].

PACAP-38 may induce migraines through various mechanisms [48]. PACAP may induce migraine attacks by activating numerous receptors across different cell types and tissues [46]. There is evidence that PACAP acts peripherally during migraine attacks. Plasma PACAP-38 levels have been found to be elevated during migraine ictal phases by several research groups [49]. Study results demonstrate that both healthy subjects and migraine patients are affected by PACAP-38 infusions, a potent vasodilator present in sensory and parasympathetic perivascular nerve fibers of the trigeminal nerve [50]. PACAP38-induced migraines are associated with sustained dilation of extracranial arteries. Activation of the PAC1 receptor may be responsible for PACAP-38-induced migraines, which could serve as a target for future anti-migraine drugs [49]. Also, studies found that PACAP38-induced headache is associated with prolonged dilatation of the MMA (middle meningeal artery) but not of the MCA (middle cerebral artery) [51]. It is unclear which PACAP-receptor causes MMA dilatation, or whether several are involved [37]. Given that migraine has a genetic background, one study showed that in patients with migraine without aura, the migraine response induced by PACAP38 infusion was not associated with either a high familial load or the presence of the rs2274316 (MEF2D) risk allele. There is no correlation between migraine response to PACAP-38 infusion and a high familial burden among migraine patients without aura [52].

Evidence for the central role of migraine mechanisms within the trigeminocervical complex comes from a study comparing VIP and PACAP-38. VIP, another peptide from the glucagon/secretin superfamily, induces headaches in healthy individuals in a manner like PACAP and causes marked dilation of cranial arteries, mediated by VPAC2 receptors, which did not coincide with activation of central trigeminovascular neurons. Only PACAP-38 caused delayed activation and sensitization of central trigeminovascular neurons, with delayed effects that induce migraine headache. These findings suggest that central, rather than dural-peripheral, mechanisms may be more relevant to migraine initiation [53]. However, unlike PACAP, VIP does not lead to delayed migraine attacks or provoke migraines in individuals prone to them. This contrast highlights that vascular dilation alone is not sufficient to account for the complex mechanisms underlying migraine pathophysiology [54].

Unlike CGRP, PACAP plays a significant role in triggering many of the premonitory symptoms of migraines, such as fatigue, yawning, neck stiffness, increased hunger or appetite, mood changes, and difficulty concentrating, as well as several accompanying features during the migraine attack itself, including photophobia, phonophobia, flushing, a sensation of heat, palpitations, and dizziness [39]. It is noteworthy that both CGRP and PACAP induced premonitory symptoms to a similar extent, not only in patients who subsequently developed migraine-like headache, but also in those in whom these symptoms were not followed by migraine. This observation raises the possibility that such manifestations may reflect peptide-induced responses rather than being specific premonitory features of migraine [39]. Premonitory symptoms in migraine are believed to be primarily caused by hypothalamic activation. The hypothalamus contains the most abundant population of PACAP-38-containing neurons [55]. Within the trigeminovascular system, it modulates nociceptive transmission. Premonitory symptoms in migraine require more research to understand epidemiological patterns and verify whether they are truly reflective of hypothalamic dysfunction [56]. The possibility of interference between the sphenopalatine and trigeminal systems is suggested, as stimulation of the sphenopalatine ganglia likely contributes to the autonomic symptoms of migraine by increasing cerebral blood flow, intracranial and extracranial vasodilation, and extravasation of dural plasma proteins in humans [39,57]. The release of neuropeptides such as PACAP-38 by parasympathetic efferent fibers from the sphenopalatine ganglion may activate the trigeminovascular system. These data suggest PACAP-38 might function primarily as a neuropeptide in the parasympathetic pathways underlying migraine [7].

Dural mast cell degranulation may sustain and amplify the activation of adjacent trigeminal meningeal nociceptors, thereby contributing to the persistence of migraine pain [58]. In vitro studies show that PACAP-38 induces dural mast cell degranulation in rats [59]. PACAP-38 infusion has been shown to trigger not only migraine attacks but also a prolonged sensation of warmth and facial flushing [60]. Previous studies have shown that PACAP-induced degranulation of meningeal mast cells is not mediated by the PAC1 receptor. Instead, the findings suggest that in both rat peritoneal and meningeal mast cells, this process involves the orphan receptor MRGPRB3 [60]. MRGPRB3, believed to be the homolog of rat MRGPRB2 and human MRGPRX2, is expressed in connective tissue mast cells, including those in the meninges [61]. MRGPRB2 plays an important role in migraine pain pathways. PACAP1-38 activation of this receptor offers an alternative treatment target for debilitating migraine and further supports the role of the innate immune system in peripheral nervous system modulation [61]. Although interspecies differences in ligand efficacy warrant cautious interpretation, the activation of MrgprB2 by PACAP1-38 supports the view that this pathway may represent a potential alternative therapeutic target in migraine and further reinforces the contribution of the innate immune system to the modulation of peripheral nociceptive signaling [61].

Hence, PACAP acts by vasodilating extracranial and meningeal arteries, modulating parasympathetic pathways (which mediate autonomic symptoms), and modulating the degranulation of meningeal mast cells. PACAP performs many biological functions both inside and outside the blood–brain barrier, making it an attractive target for antibody therapy [36].

Monoclonal antibodies (mAbs), which block CGRP, represent the most important advance in migraine treatment for decades [20]. The PACAP signaling pathway may represent a valuable therapeutic avenue to complement and enhance CGRP-targeted migraine treatments, as PACAP engages distinct, independent intracellular signaling mechanisms compared with CGRP [39].

There is now strong evidence that PACAP plays a significant role in migraine pathogenesis, leading to its emergence as a novel target for migraine treatment. Consequently, understanding PACAP’s signaling pathways may lead to the identification of new therapeutic targets of particular interest for patients who do not respond to anti-CGRP therapy [7].

The first study to evaluate plasma PACAP-38 concentrations in patients with migraine, conducted more than a decade ago, showed that levels of this peptide were significantly lower during the interictal period compared with healthy volunteers, but increased during migraine attacks compared with the attack-free period. These findings suggest the involvement of PACAP-38 in the development of migraine attacks and support its potential role as a biomarker and therapeutic target in migraine [62]. These findings were supported by subsequent in vivo studies showing that plasma PACAP38 levels may increase during spontaneous migraine attacks compared with the interictal period [63].

A feline study showed that administration of sumatriptan was associated with reduced PACAP levels and improved migraine. Lower PACAP levels were also observed interictally compared with migraine attacks, supporting PACAP and the PAC1 receptor as potential therapeutic targets in migraine [64]. A recent study of migraine patients treated with anti-CGRP monoclonal antibodies assessed baseline plasma concentrations of CGRP and PACAP-38. Higher baseline CGRP levels were associated with a poorer clinical response after 6 months, supporting CGRP as a potential biomarker for treatment stratification. In contrast, PACAP-38 levels did not influence treatment outcomes, suggesting a distinct pathophysiological role in migraine [62]. To date, available studies provide very low-certainty evidence suggesting that PACAP levels may be negatively correlated with disease duration in adults with migraine and may vary substantially across different migraine phases in both adults and children [65]. However, this hypothesis remains preliminary, as more sensitive assay methods are required before PACAP38 can be implemented in clinical practice [7].

In migraine, the PAC1 receptor is considered the likely pathophysiological target of PACAP [39]. Ab181, a rodent-specific anti-PAC1 antibody, was developed to assess its effect on nociceptive activity in the trigeminocervical complex. Immunohistochemical analysis showed binding in the trigeminal and sphenopalatine ganglia, but not in the central nervous system, suggesting a potential peripheral site of action [66]. In the subsequent clinical study, AMG 301, a human monoclonal antibody targeting the PAC1 receptor, was evaluated for migraine prevention. Subcutaneous administration of AMG 301 was not superior to placebo in reducing monthly migraine days or in achieving a ≥50% reduction in MMD in patients with episodic or chronic migraine. However, AMG 301 showed a favorable tolerability profile, with no new safety concerns identified [67]. Repeated nitroglycerin injections significantly increased PACAP and PAC1R expression in the trigeminal nucleus caudalis, while PACAP6-38 administration reduced nociceptive sensitization, c-Fos overexpression, and associated synaptic alterations [68]. Studies have suggested that inhibition of PACAP signaling by Lu AG09222 may represent a novel and potentially effective strategy for migraine prevention. Unlike therapies targeting the PAC1 receptor, Lu AG09222 acts by neutralizing the PACAP ligand itself, thereby offering a distinct therapeutic mechanism [69]. A large study evaluating LY3451838, a neutralizing monoclonal antibody targeting the PACAP ligand, did not demonstrate superior efficacy over placebo in patients with treatment-resistant chronic or episodic migraine [70]. Table 2 summarizes the main studies investigating the role of PACAP receptors in migraine treatment.

### 4.2. The PACAP Neuropeptide, Sleep and Anxiety in Migraine

There is evidence that PACAP plays an important role in the pathophysiology of primary headache disorders, with particular emphasis on migraine and cluster headaches. Clearly, there is a clinical connection between these conditions and sleep disorders [71]. The effects of PACAP administration on human sleep have not been extensively studied [72]. The effects of PACAP observed in animals (increased REM sleep) have not been reproduced in humans under the conditions described [71,73]. In the circadian system, PACAP may serve as a regulator. PACAP was found to be expressed in projections from the retina to the suprachiasmatic nucleus (SCN) and the intergeniculate leaflet (IGL) of the thalamus, two central sites that regulate circadian rhythms. PACAP has been found to reset the phase of the biological clock in the SCN via the PACAP-R1 receptor and cAMP signaling [44]. The pineal gland lies outside the blood–brain barrier (BBB) and is innervated by PACAP-immunoreactive nerve fibers, which play a role in regulating the synthesis and/or secretion of melatonin [74]. PACAP exhibits circadian, phase-dependent expression, with peak levels during the dark phase in rats. Since PACAP can stimulate melatonin synthesis and the pineal gland lacks a functional BBB, intravenous PACAP may theoretically influence sleep–wake cycles by directly modulating melatonin release [71,75].

The expression of the related PACAP receptor (PAC1R) varies during the female estrous cycle in rodents. There is evidence that the PACAP pathway regulates stress responses across species. Additionally, increasing evidence suggests that the PACAP pathway is regulated both by stress and estrogen. PACAP has been associated with depression in men and anxiety-related disorders in women [76,77]. PACAP regulates stress responses via two main pathways. A descending projection from the frontal cortex to the paraventricular nucleus, modulating CRH release, and an ascending projection from the external lateral parabrachial nucleus to protein kinase C delta (PKCδ)-type neurons in the central amygdala [77].

## 5. Astrocytes, the Link Between PACAP and Glymphatic System Dysfunction in Migraine

Studies in vitro and in vivo have shown that PACAP modulates several activities of rat astrocytes, including their proliferation, plasticity, glycogen synthesis, and gliotransmitter production. Moreover, astrocytes are important components of the glymphatic system, a perivascular network surrounding brain vessels that facilitates the movement of cerebrospinal fluid throughout the brain and into the periphery to eliminate metabolic waste. A potential target for migraine treatment may be astrocytes, which can restore changes to the glymphatic system. There is still much work to be done to determine the pathways by which the glymphatic system is modulated via astrocytes. One possible pathway would be to regulate the expression of aquaporin 4 [78].

Despite the roles of astrocytes in PACAP signaling and glymphatic function in migraine pathology, we do not yet have experimental evidence linking PACAP to the glymphatic system. While the astrocytes play a central role in PACAP signaling and glymphatic function in migraine pathology, there are few studies currently available. There is a conceptual shift if PACAP also plays a role in normalizing glymphatic dysfunction and if it drives migraine through vascular, astrocytic, and clearance mechanisms. This is still a hypothetical possibility, but it is actively being discussed. PCAP is indirectly linked to glymphatic dysfunction in migraine, but there are currently no direct human studies on this topic.

## 6. Discussions

Impairment of the glymphatic system can be reflected by a low DTI-APLS index (diffusion tensor imaging along the perivascular space) and by enlargement of the perivascular space. Due to the small sample size and the lack of a temporal association between the enlarged perivascular space and the progression of chronic migraine, the results remain controversial [79].

Another discussion could focus on the differences between rodent models and humans concerning AQP expression, polarization, and astrocytic organization in migraine-like conditions. According to rodent studies, AQP4 is highly polarized to astrocytic perivascular endfeet, and loss of this polarization is observed in disease states. In humans, AQP4 polarization appears less pronounced. Because human astrocytes are larger and more complex than those of rodents, this difference may partly be explained by their size and structural complexity. Some studies suggest that, when translating pathophysiological findings from rodents to humans, it is important to consider that AQP4 polarization in humans may be approximately one-third of that observed in mice [80]. While AQP4 polarization is thought to regulate glymphatic flow and may influence migraine-related processes, this relationship is likely more variable and context-dependent in humans. Therefore, glymphatic dysfunction in migraine may be better viewed not as an independent disturbance, but as a modulatory factor interacting with PACAP signaling, sleep, and neuroinflammation.

In this review, a hypothetical, integrative mechanism linking impaired glymphatic function to increased PACAP and CGRP levels in migraine, which may in turn increase neuroinflammation, is proposed. A combination of imaging biomarkers (DTI-APLS, increase in PVS size) and neuropeptides (CGRP and PACAP) might improve therapy selection by stratifying the patients into subtypes. Symptoms of chronic migraine can also be associated with glymphatic dysfunction. An approach that combines imaging biomarkers, serum neuropeptides, and clinical aspects can be more effective in classifying and treating patients.

Although PAC1 receptor blockade reduced central nociceptive activity in experimental models, clinical findings have been inconclusive. The Phase 2 trial of the anti-PAC1 monoclonal antibody AMG 301, as well as the study of LY3451838, an anti-PACAP neutralizing antibody, failed to demonstrate superior efficacy over placebo in migraine prevention. In contrast, Lu AG09222, a humanized anti-PACAP antibody, was associated with a reduction in monthly migraine days compared with placebo. These findings suggest that selective PAC1 receptor inhibition may be insufficient and that the involvement of additional receptors, such as VPAC1 and VPAC2, or broader neutralization of the PACAP ligand, may be required to achieve a clinically relevant therapeutic effect. Thus, the role of PAC1 receptor inhibition in migraine treatment remains under investigation. Further studies are needed to clarify better the contribution of PACAP and its receptors to migraine pathophysiology and to determine which therapeutic strategy may provide the greatest clinical benefit.

## 7. Conclusions

The disability caused by migraine at a young age and the lack of therapeutic response to some drugs have prompted studies of new possible mechanisms in this disease. As part of this review, we analyzed new potential pain-triggering mechanisms and possible glymphatic system involvement. There are at least four perspectives that can be used to study the relationship between the glymphatic system and migraine: neuroinflammation, CGRP, CSD, and sleep. Regarding every probable mechanism, we detailed every direction and presented recent data.

PACAP has also been shown to play a significant role in migraine pathogenesis, leading to its emergence as a novel migraine treatment target, and we have presented recent data on these mechanisms. Clinicians should consider this aspect as well, especially with patients who do not respond to anti-CGRP treatments.

Another hypothesis involves the interaction between PACAP and the glymphatic system, and astrocytes may play a key role in this interaction. To our knowledge, there are no in vivo or in vitro data in the literature regarding this topic. More research is needed to understand this probable mechanism, which may provide new perspectives on migraine physiology.

## Figures and Tables

**Table 1:** Peripheral and central effects of PACAP.

Aspect	Peripheral Effects	Central Effects
Main localization	Trigeminovascular system, dural nerve fibers, cerebral vessels, sphenopalatine ganglion, otic ganglia	Hypothalamus, supraoptic nucleus, trigeminal nucleus caudalis, brainstem nuclei
Main receptors	PAC1, VPAC1, VPAC2	PAC1 is widely expressed in the CNS, particularly in the hypothalamus
Vascular role	Contributes to cranial and meningeal vasodilation	More indirect role through modulation of central autonomic and nociceptive circuits
Autonomic symptoms	Activation of the sphenopalatine ganglion may contribute to cranial autonomic symptoms	The pontine superior salivatory nucleus and central autonomic circuits may contribute to autonomic manifestations
Neurogenic inflammation	May promote mast cell degranulation and release of inflammatory mediators	May modulate neuroinflammation through interactions with neurons and glial cells
Role in nociception	Peripheral antinociceptive effects	Appears to have a pronociceptive role and may contribute to central sensitization
Circadian rhythm	-	Involved in circadian entrainment through the retinohypothalamic tract and suprachiasmatic nucleus
Stress response	May contribute indirectly through peripheral neuroimmune and nociceptive interactions	PACAP/PAC1 signaling is involved in activation of the hypothalamic–pituitary–adrenal axis and stress-related circuits, including the amygdala, locus coeruleus, and raphe nuclei
Migraine-relevant structures	Dura mater, cranial vessels, cranial parasympathetic ganglia, peripheral trigeminal endings	Trigeminal nucleus caudalis, trigeminocervical complex, periaqueductal gray, locus coeruleus, raphe nuclei, hypothalamus
Overall role in migraine	Contributes to vasodilation, neurogenic inflammation, trigeminovascular activation, and autonomic symptoms	Contributes to central sensitization, pain processing, autonomic regulation, circadian control, and stress responses

**Table 2:** Studies on the role of PACAP receptors in migraine treatment.

Study	Target	Type	Main Result
Ab181—anti-PAC1 antibody in rodents [66]	PAC1 receptor	Preclinical study	Peripheral binding pattern suggests a possible extracerebral mechanism for modulating trigeminal nociception.
AMG 301—human anti-PAC1 monoclonal antibody [67]	PAC1 receptor	Phase 2 clinical trial	No benefit over placebo in migraine prevention.
The PACAP6-38 receptor antagonist [68]	VPAC2 receptor PAC1 receptor	Preclinical study	PACAP6-38 reduced nitroglycerin-induced central sensitization in the trigeminal nucleus caudalis
Lu AG09222, a humanized anti-PACAP antibody [69]	The PACAP ligand, not the receptor	Phase 2 clinical trial	Reduced monthly migraine days compared with placebo.
LY3451838, an anti-PACAP neutralizing antibody [70]	The PACAP ligand, not the receptor	Preclinical study, Phase 1 and 2 clinical trial	Did not demonstrate statistically significant superiority over placebo in patients with treatment-resistant migraine.

## Data Availability

The data are available in the cited sources.

## Abbreviations

AQP	aquaporin-4
BBB	blood–brain barrier
CGRP	calcitonin-gene related peptide
CSD	cortical spreading depression
CSF	cerebrospinal fluid
MLVs	meningeal lymphatic vessels
NE	norepinephrine
PACAP	pituitary adenylate cyclase-activating peptide
PVS	perivascular space
ROS	reactive oxygen species

## References

[1] Puledda F., Silva E.M., Suwanlaong K., Goadsby P.J. (2023). Migraine: From pathophysiology to treatment. J. Neurol..

[2] Chen Z.F., Kong X.M., Yang C.H., Li X.Y., Guo H., Wang Z.W. (2024). Global, regional, and national burden and trends of migraine among youths and young adults aged 15–39 years from 1990 to 2021: Findings from the global burden of disease study 2021. J. Headache Pain.

[3] Cuciureanu D.I., Bistriceanu C.E., Vulpoi G.-A., Cuciureanu T., Antochi F., Roceanu A.-M. (2024). Migraine Comorbidities. Life.

[4] Huang W., Zhang Y., Zhou Y., Zong J., Qiu T., Hu L., Pan S., Xiao Z. (2023). Glymphatic Dysfunction in Migraine Mice Model. Neuroscience.

[5] Kaur J., Chopp M., Jiang Q. (2024). Lymphatic System of the Brain. Reference Module in Neuroscience and Biobehavioral Psychology.

[6] Schain A.J., Melo-Carrillo A., Strassman A.M., Burstein R. (2017). Cortical Spreading Depression Closes Paravascular Space and Impairs Glymphatic Flow: Implications for Migraine Headache. J. Neurosci. Off. J. Soc. Neurosci..

[7] Guo S., Jansen-Olesen I., Olesen J., Christensen S.L. (2023). Role of PACAP in migraine: An alternative to CGRP?. Neurobiol. Dis..

[8] Vittorini M.G., Sahin A., Trojan A., Yusifli S., Alashvili T., Bonifácio G.V., Paposhvili K., Tischler V., Lampl C., Sacco S. (2024). The glymphatic system in migraine and other headaches. J. Headache Pain.

[9] Gao Y., Liu K., Zhu J. (2023). Glymphatic system: An emerging therapeutic approach for neurological disorders. Front. Mol. Neurosci..

[10] Wang D.J., Hua J., Cao D., Ho M.L. (2023). Neurofluids and the glymphatic system: Anatomy, physiology, and imaging. Br. J. Radiol..

[11] Liao J., An Z., Cheng Q., Liu Y., Chen Y., Su Z., Usman A.M., Tang Z., Xiao G. (2024). The glymphatic system: A new insight into the understanding of neurological diseases. Brain X.

[12] Wardlaw J.M., Benveniste H., Nedergaard M., Zlokovic B.V., Mestre H., Lee H., Doubal F.N., Brown R., Ramirez J., MacIntosh B.J. (2020). Perivascular spaces in the brain: Anatomy, physiology and pathology. Nat. Rev. Neurol..

[13] Yu L., Hu X., Li H., Zhao Y. (2022). Perivascular Spaces, Glymphatic System and MR. Front. Neurol..

[14] Gouveia-Freitas K., Bastos-Leite A.J. (2021). Perivascular spaces and brain waste clearance systems: Relevance for neurodegenerative and cerebrovascular pathology. Neuroradiology.

[15] Jessen N.A., Munk A.S., Lundgaard I., Nedergaard M. (2015). The Glymphatic System: A Beginner’s Guide. Neurochem. Res..

[16] Iliff J.J., Wang M., Liao Y., Plogg B.A., Peng W., Gundersen G.A., Benveniste H., Vates G.E., Deane R., Goldman S.A. (2012). A paravascular pathway facilitates CSF flow through the brain parenchyma and the clearance of interstitial solutes, including amyloid β. Sci. Transl. Med..

[17] Peng S., Liu J., Liang C., Yang L., Wang G. (2023). Aquaporin-4 in glymphatic system, and its implication for central nervous system disorders. Neurobiol. Dis..

[18] Gomolka R.S., Hablitz L.M., Mestre H., Giannetto M., Du T., Hauglund N.L., Xie L., Peng W., Martinez P.M., Nedergaard M. (2023). Loss of aquaporin-4 results in glymphatic system dysfunction via brain-wide interstitial fluid stagnation. eLife.

[19] Giannetto M.J., Gomolka R.S., Gahn-Martinez D., Newbold E.J., Bork P.A.R., Chang E., Gresser M., Thompson T., Mori Y., Nedergaard M. (2024). Glymphatic fluid transport is suppressed by the aquaporin-4 inhibitor AER-271. Glia.

[20] Troili F., Cipollini V., Moci M., Morena E., Palotai M., Rinaldi V., Romano C., Ristori G., Giubilei F., Salvetti M. (2020). Perivascular Unit: This Must Be the Place. The Anatomical Crossroad Between the Immune, Vascular and Nervous System. Front. Neuroanat..

[21] Cha M.J., Kang K.W., Shin J.W., Kim H., Kim J. (2024). Understanding the Connection between the Glymphatic System and Migraine: A Systematic Review. Headache Pain Res..

[22] Rosic B., Dukefoss D.B., Åbjørsbråten K.S., Tang W., Jensen V., Ottersen O.P., Enger R., Nagelhus E.A. (2019). Aquaporin-4-independent volume dynamics of astroglial endfeet during cortical spreading depression. Glia.

[23] Enger R., Dukefoss D.B., Tang W., Pettersen K.H., Bjørnstad D.M., Helm P.J., Jensen V., Sprengel R., Vervaeke K., Ottersen O.P. (2017). Deletion of Aquaporin-4 Curtails Extracellular Glutamate Elevation in Cortical Spreading Depression in Awake Mice. Cereb. Cortex.

[24] Yang M.F., Ren D.X., Pan X., Li C.X., Xu S.Y. (2024). The Role of Astrocytes in Migraine with Cortical Spreading Depression: Protagonists or Bystanders? A Narrative Review. Pain Ther..

[25] Toriello M., González-Quintanilla V., Pérez-Pereda S., Fontanillas N., Pascual J. (2021). The Potential Role of the Glymphatic System in Headache Disorders. Pain Med..

[26] Mikhailov N., Virenque A., Koroleva K., Eme-Scolan E., Teleman M., Abdollahzadeh A., Giniatullina R., Gafurov O., Krivoshein G., Malm T. (2022). The role of the meningeal lymphatic system in local meningeal inflammation and trigeminal nociception. Sci. Rep..

[27] Nelson-Maney N.P., Bálint L., Beeson A.L., Serafin D.S., Kistner B.M., Douglas E.S., Siddiqui A.H., Tauro A.M., Caron K.M. (2024). Meningeal lymphatic CGRP signaling governs pain via cerebrospinal fluid efflux and neuroinflammation in migraine models. J. Clin. Investig..

[28] Liu X., Wu G., Tang N., Li L., Liu C., Wang F., Ke S. (2021). Glymphatic Drainage Blocking Aggravates Brain Edema, Neuroinflammation *via* Modulating TNF-α, IL-10, and AQP4 After Intracerebral Hemorrhage in Rats. Front. Cell. Neurosci..

[29] Zhang J., Simoes R., Guo T., Cao Y.Q. (2024). Neuroimmune interactions in the development and chronification of migraine headache. Trends Neurosci..

[30] Messlinger K. (2018). The big CGRP flood—Sources, sinks and signalling sites in the trigeminovascular system. J. Headache Pain.

[31] Thomas J.L., Schindler E.A., Gottschalk C. (2024). Meningeal lymphatic vessel dysfunction driven by CGRP signaling causes migraine-like pain in mice. J. Clin. Investig..

[32] Avona A., Burgos-Vega C., Burton M.D., Akopian A.N., Price T.J., Dussor G. (2019). Dural Calcitonin Gene-Related Peptide Produces Female-Specific Responses in Rodent Migraine Models. J. Neurosci. Off. J. Soc. Neurosci..

[33] Yi T., Gao P., Zhu T., Yin H., Jin S. (2022). Glymphatic System Dysfunction: A Novel Mediator of Sleep Disorders and Headaches. Front. Neurol..

[34] Kilic K., Karatas H., Dönmez-Demir B., Eren-Kocak E., Gursoy-Ozdemir Y., Can A., Petit J.M., Magistretti P.J., Dalkara T. (2018). Inadequate brain glycogen or sleep increases spreading depression susceptibility. Ann. Neurol..

[35] Wei X., Yan J., Tillu D., Asiedu M., Weinstein N., Melemedjian O., Price T., Dussor G. (2015). Meningeal norepinephrine produces headache behaviors in rats via actions both on dural afferents and fibroblasts. Cephalalgia Int. J. Headache.

[36] Russo A.F. (2017). Overview of neuropeptides: Awakening the senses?. Headache.

[37] Edvinsson L., Tajti J., Szalárdy L., Vécsei L. (2018). PACAP and its role in primary headaches. J. Headache Pain.

[38] Jansen-Olesen I., Hougaard Pedersen S. (2018). PACAP and its receptors in cranial arteries and mast cells. J. Headache Pain.

[39] Kuburas A., Russo A.F. (2023). Shared and independent roles of CGRP and PACAP in migraine pathophysiology. J. Headache Pain.

[40] Sándor K., Bölcskei K., McDougall J.J., Schuelert N., Reglődi D., Elekes K., Pethő G., Pintér E., Szolcsányi J., Helyes Z. (2009). Divergent peripheral effects of pituitary adenylate cyclase-activating polypeptide-38 on nociception in rats and mice. Pain.

[41] Sándor K., Kormos V., Botz B., Imreh A., Bölcskei K., Gaszner B., Markovics A., Szolcsányi J., Shintani N., Hashimoto H. (2010). Impaired nocifensive behaviours and mechanical hyperalgesia, but enhanced thermal allodynia in pituitary adenylate cyclase-activating polypeptide deficient mice. Neuropeptides.

[42] Waschek J.A., Baca S.M., Akerman S. (2018). PACAP and migraine headache: Immunomodulation of neural circuits in autonomic ganglia and brain parenchyma. J. Headache Pain.

[43] Bahra A., Matharu M.S., Buchet C., Frackowiak R.S.J., Goadsby P.J. (2001). Brainstem activation specific to migraine headache. Lancet.

[44] Hannibal J., Ding J.M., Chen D., Fahrenkrug J., Larsen P.J., Gillette M.U., Mikkelsen J.D. (1997). Pituitary adenylate cyclase-activating peptide (PACAP) in the retinohypothalamic tract: A potential daytime regulator of the biological clock. J. Neurosci..

[45] Hammack S.E., May V. (2015). Pituitary adenylate cyclase activating polypeptide in stress-related disorders: Data convergence from animal and human studies. Biol. Psychiatry.

[46] Sundrum T., Walker C.S. (2018). Pituitary adenylate cyclase-activating polypeptide receptors in the trigeminovascular system: Implications for migraine. Br. J. Pharmacol..

[47] Dickson L., Finlayson K. (2009). VPAC and PAC receptors: From ligands to function. Pharmacol. Ther..

[48] Ashina H., Guo S., Vollesen A.L.H., Ashina M. (2017). PACAP38 in human models of primary headaches. J. Headache Pain.

[49] Amin F.M., Hougaard A., Schytz H.W., Asghar M.S., Lundholm E., Parvaiz A.I., de Koning P.J., Andersen M.R., Larsson H.B., Fahrenkrug J. (2014). Investigation of the pathophysiological mechanisms of migraine attacks induced by pituitary adenylate cyclase-activating polypeptide-38. Brain A J. Neurol..

[50] Schytz H.W., Birk S., Wienecke T., Kruuse C., Olesen J., Ashina M. (2009). PACAP38 induces migraine-like attacks in patients with migraine without aura. Brain A J. Neurol..

[51] Amin F.M., Asghar M.S., Guo S., Hougaard A., Hansen A.E., Schytz H.W., van der Geest R.J., de Koning P.J., Larsson H.B., Olesen J. (2012). Headache and prolonged dilatation of the middle meningeal artery by PACAP38 in healthy volunteers. Cephalalgia Int. J. Headache.

[52] Guo S., Vollesen A.L., Hansen R.D., Esserlind A.L., Amin F.M., Christensen A.F., Olesen J., Ashina M. (2017). Part I: Pituitary adenylate cyclase-activating polypeptide-38 induced migraine-like attacks in patients with and without familial aggregation of migraine. Cephalalgia Int. J. Headache.

[53] Akerman S., Goadsby P.J. (2015). Neuronal PAC1 receptors mediate delayed activation and sensitization of trigeminocervical neurons: Relevance to migraine. Sci. Transl. Med..

[54] Rahmann A., Wienecke T., Hansen J.M., Fahrenkrug J., Olesen J., Ashina M. (2008). Vasoactive intestinal peptide causes marked cephalic vasodilation, but does not induce migraine. Cephalalgia Int. J. Headache.

[55] Arimura A., Somogyvári-Vigh A., Miyata A., Mizuno K., Coy D.H., Kitada C. (1991). Tissue distribution of PACAP as determined by RIA: Highly abundant in the rat brain and testes. Endocrinology.

[56] Gollion C., De Icco R., Dodick D.W., Ashina H. (2022). The premonitory phase of migraine is due to hypothalamic dysfunction: Revisiting the evidence. J. Headache Pain.

[57] Akerman S., Holland P.R., Summ O., Lasalandra M.P., Goadsby P.J. (2012). A translational in vivo model of trigeminal autonomic cephalalgias: Therapeutic characterization. Brain A J. Neurol..

[58] Levy D., Burstein R., Kainz V., Jakubowski M., Strassman A.M. (2007). Mast cell degranulation activates a pain pathway underlying migraine headache. Pain.

[59] Baun M., Pedersen M.H., Olesen J., Jansen-Olesen I. (2012). Dural mast cell degranulation is a putative mechanism for headache induced by PACAP-38. Cephalalgia Int. J. Headache.

[60] Pedersen S.H., la Cour S.H., Calloe K., Hauser F., Olesen J., Klaerke D.A., Jansen-Olesen I. (2019). PACAP-38 and PACAP(6-38) Degranulate Rat Meningeal Mast Cells *via* the Orphan MrgB_3_-Receptor. Front. Cell. Neurosci..

[61] Sbei S., Moncrief T., Limjunyawong N., Zeng Y., Green D.P. (2023). PACAP activates MRGPRX2 on meningeal mast cells to drive migraine-like pain. Sci. Rep..

[62] Rubino E., Marcinnò A., Boschi S., Piella E.M., Chiarandon A.M., Roveta F., Gallo E., Rainero I. (2025). Plasma CGRP but not PACAP-38 concentrations are associated with response to anti-CGRP monoclonal antibodies in migraine. Confin. Cephalalgica.

[63] Tuka B., Helyes Z., Markovics A., Bagoly T., Szolcsányi J., Szabó N., Tóth E., Kincses Z.T., Vécsei L., Tajti J. (2013). Alterations in PACAP-38-like immunoreactivity in the plasma during ictal and interictal periods of migraine patients. Cephalalgia.

[64] Zagami A.S., Edvinsson L., Goadsby P.J. (2014). Pituitary adenylate cyclase activating polypeptide and migraine. Ann. Clin. Transl. Neurol..

[65] Zhu G., Wang M., Kong F. (2024). Blood serum levels of PACAP and migraine onset: A systematic review and meta-analysis of observational studies. Headache.

[66] Hoffmann J., Miller S., Martins-Oliveira M., Akerman S., Supronsinchai W., Sun H., Shi L., Wang J., Zhu D., Lehto S. (2020). PAC1 receptor blockade reduces central nociceptive activity: New approach for primary headache?. Pain.

[67] Ashina M., Doležil D., Bonner J.H., Zhou L., Klatt J., Picard H., Mikol D.D. (2021). A phase 2, randomized, double-blind, placebo-controlled trial of AMG 301, a pituitary adenylate cyclase-activating polypeptide PAC1 receptor monoclonal antibody for migraine prevention. Cephalalgia.

[68] Zhang L., Zhou Y., Yang L., Wang Y., Xiao Z. (2023). PACAP6-38 improves nitroglycerin-induced central sensitization by modulating synaptic plasticity at the trigeminal nucleus caudalis in a male rat model of chronic migraine. J. Headache Pain.

[69] Ashina M., Phul R., Khodaie M., Löf E., Florea I. (2024). A monoclonal antibody to PACAP for migraine prevention. N. Engl. J. Med..

[70] Johnson M.P., Krikke-Workel J., Patel C.N., Morin S.M., Turner P.K., Clark K.A., Donley D., Jin Y., Johnson K.W., Vincent M. (2025). Preclinical and clinical evaluation of LY3451838, a PACAP-neutralizing monoclonal antibody, in randomized, double-blind, placebo-controlled phase 1 and phase 2 studies involving healthy adults and adults with treatment-resistant migraine. Cephalalgia.

[71] Holland P.R., Barloese M., Fahrenkrug J. (2018). PACAP in hypothalamic regulation of sleep and circadian rhythm: Importance for headache. J. Headache Pain.

[72] Murck H., Steiger A., Frieboes R.M., Antonijevic I.A. (2007). Pituitary adenylate cyclase-activating peptide affects homeostatic sleep regulation in healthy young men. Am. J. Physiol. Endocrinol. Metab..

[73] Ahnaou A., Basille M., Gonzalez B., Vaudry H., Hamon M., Adrien J., Bourgin P. (1999). Long-term enhancement of REM sleep by the pituitary adenylyl cyclase-activating polypeptide (PACAP) in the pontine reticular formation of the rat. Eur. J. Neurosci..

[74] Liu W., Møller M. (2000). Innervation of the rat pineal gland by PACAP-immunoreactive nerve fibers originating in the trigeminal ganglion: A degeneration study. Cell Tissue Res..

[75] Simonneaux V., Ouichou A., Pévet P. (1993). Pituitary adenylate cyclase-activating polypeptide (PACAP) stimulates melatonin synthesis from rat pineal gland. Brain Res..

[76] Ramikie T.S., Ressler K.J. (2016). Stress-related disorders, pituitary adenylate cyclase-activating peptide (PACAP)ergic system, and sex differences. Dialogues Clin. Neurosci..

[77] Jiang S.Z., Zhang H.Y., Eiden L.E. (2023). PACAP Controls Endocrine and Behavioral Stress Responses via Separate Brain Circuits. Biol. Psychiatry Glob. Open Sci..

[78] Gliga O., Feliu-Soler A., Vila-Pueyo M. (2026). Non-neuronal targets for migraine therapy. Neurother. J. Am. Soc. Exp. Neurother..

[79] Cho J.W., Lee G.H., Kim J. (2026). Chronic Migraine Is Associated with Region-Specific High-Grade Enlarged Perivascular Spaces: Retrospective Matched Case-Control Study. J. Clin. Neurol..

[80] Eidsvaag V.A., Enger R., Hansson H.A., Eide P.K., Nagelhus E.A. (2017). Human and mouse cortical astrocytes differ in aquaporin-4 polarization toward microvessels. Glia.

